# Acute and Chronic Effects of Drop-Set Training: A Meta-Analysis and Systematic Review

**DOI:** 10.1186/s40798-026-01012-1

**Published:** 2026-04-01

**Authors:** Tim Havers, Florian Micke, Stephan Geisler, Steffen Held

**Affiliations:** 1 Faculty of Sport and Health Sciences, Technical University of Munich, Munich, Germany; 2 Department of Fitness and Health, IST University of Applied Sciences, Düsseldorf, Germany; 3 Department of Sport and Management, IST University of Applied Sciences, Düsseldorf, Germany

**Keywords:** Drop-set training, Resistance training, Hypertrophy, Strength, Metabolic stress, Neuromuscular fatigue, Perceived exertion, Time-efficient training, Muscle adaptation, Training intensity

## Abstract

**Background:**

Drop-set training (DROP) is a time-efficient resistance training method for hypertrophy and strength. Its long-term adaptations remain debated, particularly in relation to its acute physiological responses such as metabolic stress and fatigue. This meta-analysis examines both acute and chronic effects of DROP to provide a comprehensive evaluation of its efficacy.

**Methods:**

A systematic search was conducted across PubMed, Web of Science, SCOPUS, and SPORTDiscus up to January 20, 2026, following PRISMA guidelines. Studies comparing DROP and traditional resistance training (TRAD) on hypertrophy, strength, metabolic stress, fatigue, and perceived exertion were included. Data extraction and risk of bias assessment were performed using the PEDro scale. Meta-analyses were conducted using a random-effects model.

**Results:**

The meta-analysis, based on 12 studies (*n* = 274 participants), revealed significant increases in ratings of perceived exertion (SMD = 1.62, 95% CI [0.33 to 2.91]) and lactate levels (SMD = 0.67, 95% CI [0.20 to 1.14]) for DROP. A trend in favor of DROP was observed for heart rate, although this did not reach statistical significance (SMD = 0.45, 95% CI [− 0.12 to 1.02]). No significant differences were observed between DROP and TRAD for chronic hypertrophy (SMD = 0.04, 95% CI [− 0.29 to 0.36]), strength (SMD = − 0.04, 95% CI [− 0.34 to 0.26]), or muscle endurance adaptations (SMD = 0.53, 95% CI [− 0.20 to 1.26]).

**Conclusion:**

DROP offers a time-efficient alternative to TRAD, yielding comparable long-term gains in muscle hypertrophy and strength. Based on current evidence, DROP acutely induces significantly higher perceived exertion and lactate responses, whereas heart rate shows no consistent differences between methods. Practitioners should consider these elevated perceptual demands and potential recovery needs when integrating DROP into long-term training periodization.

## Key Points

- Drop-set training induces significantly higher perceived exertion, blood lactate concentrations, and neuromuscular fatigue than traditional resistance training.
- Both training modalities lead to comparable long-term gains in muscle hypertrophy, strength, and muscle endurance.
- While drop-set training is a highly time-efficient alternative, its elevated perceptual and neuromuscular demands might necessitate strategic recovery management to maintain long-term training adherence and frequency.

## Introduction

Resistance training (RT) is a cornerstone of physical fitness, offering a range of physiological and psychological benefits. It enhances muscular strength, power, and endurance while promoting skeletal muscle hypertrophy through the accumulation of contractile and structural proteins [1, 2]. These adaptations are driven by mechanical tension and metabolic stress, which activate anabolic signaling pathways essential for muscle protein synthesis [3–5]. Beyond athletic performance, RT improves metabolic health, reduces the risk of chronic diseases such as type 2 diabetes and cardiovascular disorders, and mitigates sarcopenia, the age-related loss of muscle mass [6, 7]. It also supports bone density, joint health, and functional mobility, making it integral to rehabilitation and general health maintenance [8]. Psychological benefits include reduced symptoms of depression and anxiety, improved mood, and greater self-esteem [9].

Resistance training encompasses a variety of techniques aimed at improving strength, hypertrophy, and endurance. Among these, advanced methods such as drop-set training (DROP) have gained traction for their potential to optimize muscle hypertrophy while minimizing training time [10–13]. DROP is characterized by performing an exercise to momentary muscular failure, immediately reducing the load, and continuing without rest until failure is reached again [14]. This approach allows for a high training volume to be accumulated within a condensed timeframe, making it particularly advantageous for individuals with limited time for exercise [15]. Beyond their long-term physiological benefits, DROP also induces pronounced acute responses, including heightened metabolic stress, increased ratings of perceived exertion (RPE), and neuromuscular fatigue [12, 16]. These acute effects are essential, as they not only contribute to long-term adaptations but also play a critical role in exercise adherence and overall performance.

Despite the theoretical advantages, the efficacy of DROP compared to traditional resistance training (TRAD) remains insufficiently explored. While existing meta-analyses offer valuable insights, they also reveal significant gaps. Sødal et al. [14] reported that both DROP and TRAD led to significant muscle hypertrophy, with no substantial differences between the methods. Notably, DROP protocols required only half to one-third of the training time compared to TRAD, emphasizing their time efficiency. Similarly, Coleman et al. [15] concluded that DROP and TRAD elicit similar muscular adaptations in terms of strength and muscle hypertrophy.

However, while these previous syntheses [14, 15] provide important insights into chronic adaptations, a critical gap remains regarding the acute physiological and perceptual demands of DROP. To date, acute responses such as lactate accumulation, heart rate, RPE, and neuromuscular fatigue have not been systematically meta-analyzed. These markers are mechanistically significant, as heightened metabolic stress and fatigue are not only potential drivers of anabolic signaling [5] but also determine the practical feasibility and psychological tolerance of a training protocol [17].

Furthermore, a re-evaluation of the chronic effects is warranted, given that the evidence base was limited at the time of previous publications (five studies in each meta-analysis [14, 15]). Since their data collection, the volume of available literature has grown, providing a more robust synthesis of chronic effects. By integrating these new data points with a first-of-its-kind analysis of acute effects, this study moves beyond the question of whether DROP works, and instead explores the physiological ‘price’ (in terms of fatigue and effort) paid for its time-efficiency. A better understanding of these immediate effects is crucial for developing evidence-based protocols that optimize both short-term performance and long-term health outcomes.

Addressing these gaps, this study aims to conduct a comprehensive meta-analysis comparing the effects of DROP and TRAD on both strength and hypertrophic adaptations, alongside their acute physiological responses. By integrating the latest research, we seek to provide a more nuanced understanding of how DROP influences muscle performance relative to established training protocols. Ultimately, this study will contribute to refining recommendations that balance training effectiveness with practical sustainability.

## Methods

### Search and Screening Procedures

This meta-analysis was conducted in accordance with the Preferred Reporting Items for Systematic Reviews and Meta-Analyses (PRISMA) guidelines [18] was pre-registered in PROSPERO (CRD42025640006). The literature search and screening processes were independently performed by two researchers (TH and SH). Four electronic databases (PubMed, Web of Science, Scopus, and SPORTDiscus) were systematically screened from inception up to January 10th, 2025, with an update performed on January 20th, 2026. Relevant search terms were combined using Boolean operators (AND/OR). The literature search was performed using the following search syntax: “Drop set” OR “Drop set training” OR “Drop set method” OR “Drop-set” OR “Descending sets” OR “Breakdown sets.” This search strategy was adapted from previous meta-analyses on DROP [14, 15]. Additionally, citation tracking and manual searches of references in primary articles and reviews were conducted to identify further relevant studies. Duplicates were removed, and the remaining studies underwent a multistage manual screening process. This included an initial review of titles, followed by abstracts, and finally, full-text evaluation of potentially eligible articles. Final inclusion or exclusion decisions were made through consensus between the two researchers (TH and SH).

The inclusion criteria were developed using the PICOS framework: (1) Population (P): Studies included healthy adults. Participants with cognitive, neurological, orthopedic, or cardiovascular conditions that could affect physical testing or performance were excluded. (2) Intervention (I): Interventions consisted of resistance training protocols employing a drop-set approach. For acute studies, protocols could involve single-session interventions assessing immediate outcomes such as metabolic stress, fatigue, or perceived exertion. Chronic studies required a minimum duration of 6 weeks or at least 8 resistance training sessions, focusing on long-term adaptations like strength and hypertrophy. (3) Comparators (C): The comparator was an active intervention involving traditional resistance training. (4) Outcomes (O): Studies needed to report at least one acute outcome (e.g., metabolic stress, fatigue markers, or perceived exertion) or one chronic outcome (e.g., muscle hypertrophy or strength-related outcomes such as one-repetition maximum (1RM) or muscle cross-sectional area). (5) Study Design (S): Only prospective, controlled two- or multi-arm intervention studies with a pre-post design were included. Full-text articles published in English in peer-reviewed journals were eligible for inclusion. The exclusion criteria were additional nutrition-based supplements were used; or resistance intervention were combined with blood flow restriction, electric muscle stimulation or similar approaches. For acute studies, those lacking detailed reporting of acute response measures (e.g., lactate levels, perceived exertion scales, or electromyography) were not included in the meta-analysis. Studies that could not be included in the meta-analysis were evaluated as part of a systematic review, ensuring comprehensive analysis of all relevant research findings.

### Assessment of Methodological Quality of the Studies

The methodological quality, including the risk of bias, of the included studies was independently assessed by two researchers (TH and SH) using the Physiotherapy Evidence Database (PEDro) scale [19]. The PEDro scale evaluates methodological rigor across 11 dichotomous items (yes/no). Criteria 2–9 assess aspects of randomization and internal validity, while criteria 10–11 evaluate the presence of statistically replicable results. Criterion 1 pertains to external validity but is not included in the calculation of the overall PEDro score. A PEDro score ranging from 0 to 10 is used to quantify study quality, with scores of ≥ 6 indicating high-quality studies [19]. This threshold ensures that included studies meet acceptable standards for methodological robustness and reliability in their findings. Discrepancies between the two researchers were resolved through discussion to ensure consistency and accuracy in the evaluation process.

### Data Extraction

Relevant data required for calculating effect sizes were independently extracted by two researchers (TH and SH) using a standardized Excel spreadsheet adapted from the Cochrane Collaboration guidelines [20]. Extracted data included means and standard deviations for pre- and post-test scores, as well as the number of participants in each group. When these values were not reported in the full-text articles, the means and pooled within-group standard deviations of change scores were used. If necessary, the study authors were contacted up to three times to request missing data. For studies that presented data only in graphical form, the WebPlotDigitizer Version 4.0 (Free Software Foundation, Boston, MA, USA) was utilized to extract means and standard deviations from figures [21]. In addition to effect size data, key study characteristics were recorded, including author, year of publication, number of participants, sex distribution, intervention details (duration, frequency, session length, and intervention type), and PEDro scale scores. This systematic approach ensured the comprehensive and consistent extraction of relevant data for meta-analytic calculations.

### Statistical Analysis

The standardized mean difference (SMD) and corresponding 95% confidence intervals (CIs) were calculated as measures of treatment effectiveness. SMD values were interpreted as follows: “trivial” (≤ 0.20), “moderate” (0.21–0.50), “large” (0.51–0.80), and “very large” (> 0.80) [22]. The SMD was coded such that positive values indicate higher outcomes for DROP compared with TRAD. Separate meta-analyses were conducted for acute and chronic effects, with additional subgroup analyses performed to explore key moderators. These subgroups included training status (trained vs. untrained participants), training volume (volume-equated vs. non-volume-equated protocols), body region (upper vs. lower body outcomes). For each meta-analysis, we calculated both a fixed effect model and a random effects model, with the random effects model considered the primary analysis to account for variability within and between studies [23]. Heterogeneity was assessed using the I² statistic, interpreted as low (≤ 50%), moderate (50–75%), or high (> 75%) heterogeneity [24]. To identify potential undue influence from individual studies, a leave-one-out analysis was conducted, where each study was sequentially removed, and the overall intervention effect and CIs were re-estimated. This process ensured the robustness of the findings. Publication bias was examined through visual inspection of funnel plot asymmetry and calculation of trim-and-fill estimates [25]. Additionally, Egger’s regression test was conducted for each meta-analysis to statistically assess funnel plot asymmetry and potential small study effects [26]. All statistical analyses and visualizations were performed using R software (version 4.1.1; The R Foundation for Statistical Computing) with the ‘meta’ package [27].

## Results

### Study Selection

**Fig. 1:**
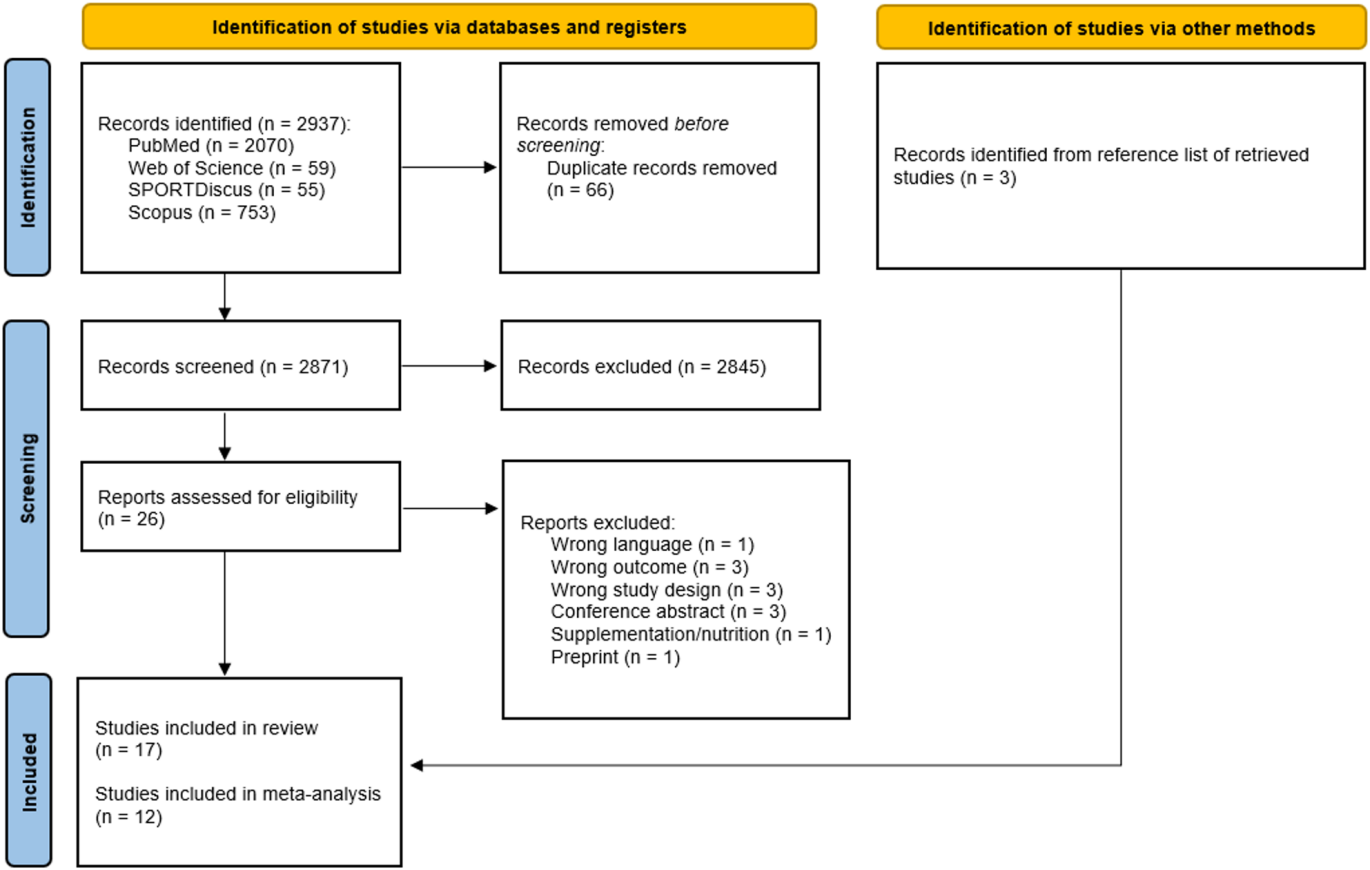
PRISMA flow chart of the search process [41]

The initial search yielded a total of 2937 records. After removing duplicates, 2871 unique records remained. These were screened based on their titles and abstracts, resulting in the exclusion of 2845 studies that did not meet the inclusion criteria. A total of 26 studies were considered potentially eligible for full-text review. Following a detailed evaluation of the full texts, 12 studies were excluded for reasons such as e.g., inappropriate study design, insufficient outcome data. Ultimately, 14 studies met the inclusion criteria and were included in the review. In addition, citation tracking and reference list reviews identified 3 additional studies that satisfied the eligibility criteria. This process resulted in a final sample of 17 studies [10–12, 16, 28–40]. However, we excluded one study [10] from further data analysis, as its dataset overlapped with that of Angleri et al. [28]. Furthermore, two studies by Goto et al. [39, 40] were excluded from the meta-analytical pooling due to their modified protocols (multiple traditional sets followed by a single drop set after 30 s of rest), which deviated significantly from standard drop-set definitions. Consequently, while these studies were reviewed systematically to provide a comprehensive overview of physiological responses, they were not included in the quantitative meta-analysis. Figure 1 presents the PRISMA flowchart summarizing the study selection process.

### Study Characteristics

Of the 16 included studies, 12 were deemed eligible for further analysis within the meta-analytical approach. Among these, 4 studies investigated acute responses [12, 34, 35, 37], while 9 studies focused on chronic adaptations [11, 12, 28, 30–33, 36, 38]; notably, Fink et al. [12] implemented both an acute and a chronic approach. These included studies for the meta analytical approach comprised a total of 274 participants, with sample sizes ranging from 9 [36] to 36 [38] and an average of 21 ± 8 participants per study. Among the participants, 8 studies included only men [11, 12, 28, 31, 32, 34–37], while 2 studies included only women [30, 33]. The participants’ ages ranged from 19.2 [32] to 35.8 years [38], with average body mass of 73.49 ± 8.1 kg, respectively. The duration of the chronic interventions varied between 6 [12, 33] and 12 weeks [28, 30, 38], with an average of 9.1 ± 2.2 weeks. Chronic training protocols included both DROP and traditional resistance training TRAD approaches, focusing on strength, local muscle endurance and hypertrophy outcomes. Acute training studies focused on primary lactate, heart rate and RPE. Most studies employed resistance-trained individuals, while 3 studies involved untrained participants [30, 32, 36]. Training volume was equated in 8 studies [11, 12, 28, 30–32, 34, 37, 38], while 3 studies did not equate volume load [33, 35, 36]. Strength outcomes were measured using 1RM (*n* = 5) [11, 28, 33, 34, 36] and XRM testing (*n* = 2) [12, 32]. Outcomes related to hypertrophy were assessed using various methods, including B-mode ultrasonography based muscle thickness (*n* = 3) [11, 30, 32], muscle cross sectional area (*n* = 3) [12, 28, 36], and other techniques such as lean body mass analysis via bioelectrical impedance analysis (*n* = 1) [38].

**Table 1:** Study characteristics

Study	Sample	Type	Intervention	Outcome	PEDro
Vilaça-Alves et al. [30]	18 untrained women (a maximum of 3 months of strength training experience) (100% females, 21.6 ± 2.9 yrs, 63.3 ± 4.7 kg body mass)	Chronic (12 weeks)	DROP: Four sets of 3 blocks of 10 repetitions at 75%, 55% and 35% of 1RM, twice a week, 120 s rest between sets; TRAD: 8 sets of 11 repetitions at 75% of 1RM. Both conditions used the biceps curl exercise seated in the Scott bench with dumbbells	Primary: • Hypertrophy: MT assessment (50% and 60% distal between the lateral epicondyle of the humerus and acromion process of the scapula)	5
Izquierdo and Marqués-Jiménez [33]	25 female basketball players (100% females, 22.6 ± 3.7 yrs, 66.4 ± 8.2 kg body mass)	Chronic (6 weeks)	DROP: 6 weeks, 2x/week, exercises: deep barbell squat, 45° leg press, seated knee extension, stiff-legged deadlift, seated knee flexion. 3 × 6 repetitions at 75% 1RM, then 3 × 10 repetitions at 55% 1RM (20 s intra-set rest, 2 min between sets) TRAD: 6-week traditional strength training, 2x/week (12 sessions). Exercises: deep barbell squat, 45° leg press, seated knee extension, stiff-legged deadlift, seated knee flexion, 3 × 12 repetitions at 70% 1RM (2 min rest between sets)	Primary: • Strength: 1RM half squat	3
Enes et al. [11]	18 trained men (0% females, 22.8 ± 3 yrs, 80.4 ± 7.2 kg body mass, resistance training experience: > 4.4 ± 0.7 yrs)	Chronic (8 weeks)	DROP: Resistance training was performed twice per week for 8 weeks. The protocol included 3 sets of 10 repetitions at 75% 1RM, followed by 6 repetitions at 55% 1RM (drop set), with a 120-s rest between sets. Exercises: barbell back squat, 45º leg press, seated knee extension, stiff-leg deadlift and seated knee flexion TRAD: Resistance training was performed twice per week for 8 weeks. The protocol consisted of 4 sets of 12 repetitions at 70% 1RM, with a 120-s rest between sets. Exercises: barbell back squat, 45º leg press, seated knee extension, stiff-leg deadlift and seated knee flexion	Primary: • Strength: 1RM squat • Hypertrophy: MT assessment of proximal (30%), middle (50%) and distal (70%) portions of lateral thigh (combination of the vastus lateralis and vastus intermedius) (distance between the greater trochanter and lateral condyle of the femur)	6
Enes et al. [31]	24 resistance training experienced males (0% females, 22.8 ± 2 yrs, 79.4 ± 8.2 kg body mass, resistance training experience: 5.1 ± 1.6 yrs)	Acute	DROP: Drop-set training consisted of three sets of 10 repetitions at 70% 1RM, followed by six additional repetitions at 50% 1RM. To ensure equal total training volume, only two sets of the 45° leg press were performed. A 2-min rest interval was maintained between all sets and exercises. Participants adhered to a movement tempo of 2:0:1:0 (2 s eccentric, 1 s concentric, no transition pauses), with set duration recorded using a timer. Exercises: barbell back squat, 45° leg press, and seated knee extension TRAD: Traditional resistance training consisted of three sets of 10 repetitions at 70% 1RM. A 2-min rest interval was maintained between all sets and exercises. Participants adhered to a movement tempo of 2:0:1:0. Exercises: barbell back squat, 45° leg press, and seated knee extension	Primary: • RPE: session RPE 10, 15, 20, 30 min after exercise • Psychophysiological response: session feeling of displeasure 15 min, 30 min	3
Enes et al. [34]	18 trained men (0% females, 23.4 ± 3.4 yrs, 80.2 ± 7.6 kg body mass; resistance training experience: 5.1 ± 1.7 years)	Chronic (8 weeks)	DROP: 8-week program, performed twice per week. Each set consisted of 10 repetitions at 75% 1RM, followed by 6 repetitions at 55% 1RM, with no intra-set rest (3 sets of 16 repetitions). Exercises included barbell back squat, 45° leg press, knee extension, stiff-legged deadlift, and knee flexion, with the drop-set method applied to the squat, leg press, and knee extension. Rest intervals of 2 min between sets and exercises. Repetition tempo: 2:0:1:0 (2 s eccentric, 1 s concentric, no transition pauses) TRAD: 8-week traditional resistance training program, performed twice per week. The protocol included 4 sets of 12 repetitions at 70% 1RM for the squat, 45° leg press, and knee extension. One additional set per exercise was performed, with a 5% reduction in load. Rest intervals of 2 min between sets and exercises. Repetition tempo: 2:0:1:0	Primary: • Strength: 1RM leg press • Muscle endurance: localized muscular endurance (leg press) Secondary: • Psychophysiological response: session RPE, monotony, and strain	6
Enes et al. [37]	15 resistance trained males (29.7 ± 6.1 yrs, 83.4 ± 7.6 kg body mass, resistance training experience: 11.4 ± 6.7 yrs)	Acute	DROP: The drop-set method consisted of 10 initial repetitions at 70% 1RM, followed by 10 additional repetitions at 50% 1RM, with a minimal intraset rest interval (< 5 s) in the barbell bench press TRAD: The traditional method involved 3 sets of 20 repetitions at 60% 1RM for the barbell bench press	Primary: • Blood lactate Secondary: • Aerobic EE whole session, absolute aerobic EE, relative aerobic EE, aerobic V·O2 • Anaerobic EE whole session, absolute anaerobic EE, relative anaerobic EE, anaerobic V·O2	3
Angleri et al. [28]	31 resistance training experienced males (0% females, 27 ± 4 yrs, 84.6 ± 8.6 kg body mass, resistance training experience: 6.4 ± 2.0 yrs)	Chronic (12 weeks)	DROP: Participants completed 3–5 sets of leg press and knee extension, with a 2-min rest between sets and exercises. Sets were performed to concentric failure at ~ 50–75% 1RM. Training was conducted twice per week for 12 weeks TRAD: Participants completed 3–5 sets of leg press and knee extension, with a 2-min rest between sets and exercises. The protocol consisted of 6–12 repetitions at 75% 1RM. Training was conducted twice per week for 12 weeks	Primary: • Strength: 1RM leg press & 1RM leg extension • Hypertrophy: CSA of M. vastus lateralis	6
Silva et al. [35]	16 trained men (0% females, 32 ± 10.5 yrs, resistance training experience: >3 yrs)	Acute	DROP: Drop-set training began at 80% 1RM, with repetitions performed until concentric failure. The load was then reduced to 60% and 40% 1RM, respectively. Three sets were performed with a 1:30 min rest between sets. Load reduction was facilitated by two evaluators to minimize rest time. Exercises: squat, leg curl bench, seated leg extension and the plantar flexion TRAD: Traditional training was performed at 70% 1RM until concentric failure for three sets, with a 1:30 min rest between sets. Exercises: squat, leg curl bench, seated leg extension and the plantar flexion	Primary: • Blood lactate • HR pre to post training; • RPE: session RPE Omni Secondary: • Psychophysiological response: Borg scale for pain (post training, 24 h post training)	3
Varović et al. [32]	16 physically active men (not trained) (19.2 ± 1.1 yrs, 78.5 ± 7.1 kg body mass)	Chronic (8 weeks)	DROP: The program consisted of leg extension exercises with a progressively increasing number of sets each week, following a linear periodization model. Training loads were adjusted based on individual progression. The starting leg alternated each session to balance performance. After completing repetitions on one leg, participants immediately switched to the other before resting for 120 s. The cadence was 1 s concentric and 2 s eccentric. Sets were performed to momentary muscle failure at ~5RM, followed by two load reductions (− 20% and − 10–15%) with continued repetitions until MMF. Target repetition range was 3–7, with loads adjusted accordingly. Training started with one weekly session (3 sets), progressing to two sessions (4 sets per session) in Week 2 TRAD: The program followed the same structure as DROP, with leg extension exercises and progressive set increases under a linear periodization model. Participants performed repetitions at ~ 15RM to muscle failure, targeting a range of 13–17 repetitions, with load adjustments to maintain this range. Training started with one weekly session (3 sets), increasing to two sessions (4 sets per session) in Week 2. From Week 3 onward, participants trained three times per week, peaking at 15 sets in Week 7	Primary: • Strength: e1RM leg extension, knee extension PTQ and ATQ • Hypertrophy: MT assessment (M. rectus femoris & M. vastus lateralis at 30%, 50% & 70% along the muscle length)	6
Costa et al. [16]	18 male young adults with previous experience in resistance training (0% females, 21.5 ± 2.4 yrs, 77.8 ± 7.1 kg body mass)	Acute	DROP: In the DROP condition, participants performed 2 sets of 10 repetitions at 12RM, followed by a decrease in intensity to 15RM with no rest interval, and 5 additional repetitions were performed. A 6-min rest interval was included between sets. Exercise: bench press, leg press TRAD: In the TRAD condition, participants performed 3 sets of 10 repetitions at 12RM with 3 min of rest between sets. Exercise: bench press, leg press	Secondary: • Neuromuscular responses: CMJ pre to post training, peak force pre to post training, peak power pre to post training	6
Angleri et al. [29]	12 participants with at least 4 months of resistance training experience (23 ± 2 yrs, 79.8 ± 5.9 kg body mass)	Acute	DROP: Each leg was randomized to either the DROP or TRAD intervention. In the DROP condition, each set began with 75% 1RM, with repetitions performed until concentric failure. After a brief rest (~ 15s), the load was reduced by ~ 20%, and additional repetitions were performed until failure. Another 15s rest allowed for a further 20% reduction, and more repetitions were completed until failure. This process was repeated for a total of three sets, with 2 min of rest between sets TRAD: Each leg was randomized to either the DROP or TRAD intervention. The TRAD protocol involved 3 sets of leg extension with a 2-min rest between sets, performing 10 repetitions at 75% 1RM for each set	Secondary: • Microvascular oxygenation: total area under the curve of the entire session for tHb, stO2, HbO2, HHb, HbDiff; • Muscle activity: Normalized EMG (%MVIC) amplitude of M. vastus medialis	3
Fink et al. [12]	16 active male college students with recreational experience in strength training participated in the study. Subjects had not engaged in regular training for more than 1 year prior to the experiment (0% females, 22.2 ± 2.9 yrs, 66.4 ± 7.6 kg body mass)	Acute	DROP: The DROP group performed a single set starting with a load at 12RM, reducing the load by 20% each time failure was reached consecutively three times. The number of repetitions varied after each load reduction, with participants performing as many repetitions as possible. Staff adjusted the weight stack pin to minimize rest time between load changes, maximizing continuous time under tension. Exercise: Cable triceps push-down TRAD: The TRAD group performed 3 sets to failure at 12RM, with 90 s of rest between sets. Participants were instructed to perform each repetition with a fast concentric movement (1 s) and a slow eccentric movement (2 s). Exercise: Cable triceps push-down	Primary: • Lactate: post 0, 2 & 5 min • HR: pre to post exercise • RPE: after a single bout of resistance training Secondary: • MT assessment: long head of the M. triceps brachii after a single bout of resistance training • Neuromuscular responses: Maximal voluntary contraction after a single bout of resistance training	6
Fink et al. [12]	16 active male college students with recreational experience in strength training participated in the study. Subjects had not engaged in regular training for more than 1 year prior to the experiment (0% females, 22.2 ± 2.9 yrs, 66.4 ± 7.6 kg body mass)	Chronic (6 weeks)	DROP: The DROP group performed a single set starting with 12RM, with the load reduced by 20% after each instance of failure, which occurred 3 times consecutively. The number of repetitions was not fixed after each reduction, and participants performed as many repetitions as possible. A staff member adjusted the weight stack pin to minimize time loss between load changes and maximize continuous time under tension. Exercise: Cable triceps push-down TRAD: The TRAD group performed 3 sets to failure at 12RM, with 90 s of rest between sets. Participants were instructed to perform each repetition with a fast concentric movement (1 s) and a slow eccentric movement (2 s). Exercise: Cable triceps push-down	Primary: • Strength: 12RM in lbs cable triceps push-downs • Hypertrophy: muscle CSA (M. triceps brachii pre to post exercise)	6
Ozaki et al. [36]	9 untrained young men (0% females, mean ± SE: 26 ± 1 yrs, 65.1 ± 2.5 kg body mass)	Chronic (8 weeks)	DROP: Each arm was randomized into one of three groups. The DROP group performed a single high-load set at 80% 1RM, followed by four drop sets at 65%, 50%, 40%, and 30% 1RM, without recovery intervals between sets. Each set was performed until concentric failure, with concentric contractions as fast as possible (~ 1 s) and eccentric contractions lasting 2 s, guided by a metronome. Dumbbells for each load were prepared before the session, and the weights were exchanged within 5 s after each failure. Exercise: dumbbell curl TRAD: Each arm was randomized into one of three groups. The TRAD group performed 3 sets of high-load resistance exercise at 80% 1RM. Each set was performed until concentric failure, with concentric contractions at ~ 1 s and eccentric contractions at 2 s, using a metronome. The recovery interval between sets was 3 min. Exercise: dumbbell curl	Primary: • Strength: Isometric strength • Hypertrophy: Elbow flexor muscle CSA • Muscle endurance: elbow flexor at 30%1RM	5
Fisher et al. [38]	36 participants with at least 6 months of resistance training experience (35.8 ± 11.1 yrs, 72.2 ± 13.8 kg body mass)	Chronic (12 weeks)	DROP: Training was conducted twice a week (with at least 48 h between sessions) for 12 weeks. Each exercise was performed for one set per session with a 2:4 repetition duration until MMF. The program consisted of two sessions: Workout A (chest press, leg press, pull-down, overhead press, adductor, abductor, abdominal flexion, and lumbar extension using bodyweight or manual resistance) and Workout B (pec-fly, pullover, leg extension, dip, biceps curl, seated calf raise, leg curl, and core torso rotation). All groups performed a single set of each exercise for both workouts. However, the DROP method was applied only to the chest press, leg press, and pull-down exercises in Workout A. These exercises were performed to MMF, with the load increased by ∼5% once participants could exceed 12 repetitions. In the DROP condition, participants first performed 8–12 repetitions to MMF, then reduced the load by ∼30% and continued to MMF. The HLDROP used heavier loads for 4 repetitions to MMF, reduced the load by ∼20%, and continued to MMF before reducing again by another 20% TRAD: Training was conducted twice a week (with at least 48 h between sessions) for 12 weeks, with each exercise performed for one set per session, using a 2:4 repetition duration until MMF. The program followed two sessions: Workout A (same exercises as in DROP) and Workout B (same exercises as in DROP). All exercises were performed to MMF with loads permitting 8–12 repetitions. When participants could exceed 12 repetitions, the load was increased by ∼5%. The TRAD group performed all exercises with no drop set, following the same load and repetition range as the DROP and HLDROP groups, which ensured parity in training load and repetition volume between the groups	Primary: • Hypertrophy: LBM	7
Goto et al. [40]	8 physically active individuals (20–23 yrs; 71.5 kg ± 2.0 [SEM] body mass, recreationally experienced with resistance training	Acute	TRAD: Resistance training consisting of five sets at 90% 1RM with 3-min rests. Leg extension exercise DROP: The study implemented three different DROP protocols. Each protocol consisted of five sets of TRAD, followed by 30 s of rest and a subsequent set of DROP (DROP with 50%, 70% or 90% 1RM) Each subject performed each protocol	Secondary: • Change in lactate concentration (measured pre-exercise, every 1–5 min, 7, 10, 12, 15, 30, and 60 min post-exercise) • Change in thigh girth (measured pre-exercise and immediately post-exercise) • Change in MVIC (measured pre-exercise and immediately post-exercise)	4
Goto et al. [39]	17 active male subjects (19–22 yrs) with no prior experience in resistance training	Chronic (10 weeks)	Participants performed leg presses and leg extensions twice a week for 10 weeks. A modified DROP training program was implemented for the first six weeks, consisting of the following: the first set at 80% 1RM, followed by a 30-s rest; the second set at 60% 1RM, followed by a 30-s rest and a drop set at 40% 1RM; a 3-min rest; and a similar scheme involving 70%, 50% and 40% 1RM, followed by a further 3-min rest and a final scheme involving 60% 1RM, 50% 1RM and 40% 1RM For the final 4 weeks, nine subjects were assigned to TRAD (five sets at 90% 1RM with a 3-min rest period) and eight subjects to DROP (five sets of TRAD followed by a 30-s rest period and a sixth set at 50% 1RM)	Secondary: • Change in CSA (quadriceps femoris) • Change in 1RM • Change in MVIC • Change in RFD	5

*1RM* one-repetition maximum, *e1RM* estimated one-repetition maximum, *MT* muscle thickness, *CMJ* countermovement jump, *CSA* cross-sectional area, *DROP* drop-set training, *HLDROP* heavy-load drop-set training, *tHb* total hemoglobin, *stO2* tissue saturation index, *HbO2* oxygenated hemoglobin, *HHb* deoxygenated hemoglobin, *HbDiff* hemoglobin difference, *EE* energy expenditure, *EMG* electromyography, *HR* heart rate, *MMF* momentary muscle failure, *MT* muscle thickness, *MVC* maximal voluntary contraction, *RPE* rating of perceived exertion, *TRAD* traditional resistance training, *V·O2* oxygen consumption, *PTQ* peak torque, *ATQ* average torque, *LBM* lean body mass, *RFD* rate of force development

### PEDro Scale Assessment

The methodological quality of the included studies, assessed via the PEDro scale, ranged from 3 to 7, with an average score of 4.8 ± 1.4. Studies scoring ≥ 6 were classified as high quality, with 7 studies falling into this category. Detailed descriptions of the training protocols, populations, and outcomes are provided in Table 1, offering a comprehensive overview of the study designs and methodologies included in this meta-analysis.

### Meta-analytical Findings on Acute Responses

**Fig. 2:**
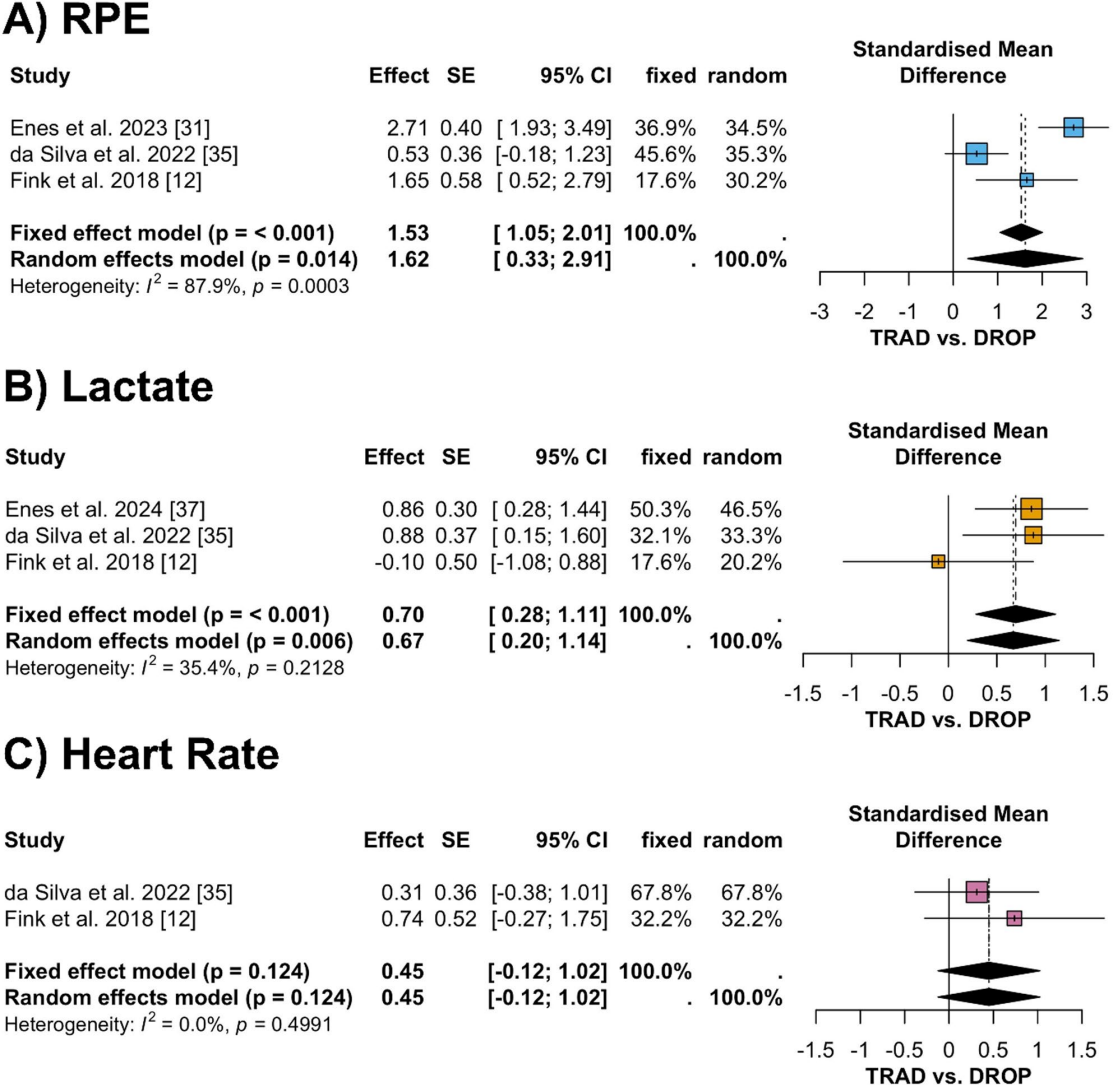
Forest plots displaying the standardized mean differences and 95% confidence intervals for acute perceptual and physiological responses, comparing drop-set versus traditional resistance training. Panels represent (**A**) rating of perceived exertion, **B** blood lactate concentration, and **C** heart rate. *CI* confidence interval, *DROP* drop-set training, *RPE* rating of perceived exertion, *SMD* standardized mean difference, *TRAD* traditional resistance training

**Fig. 3:**
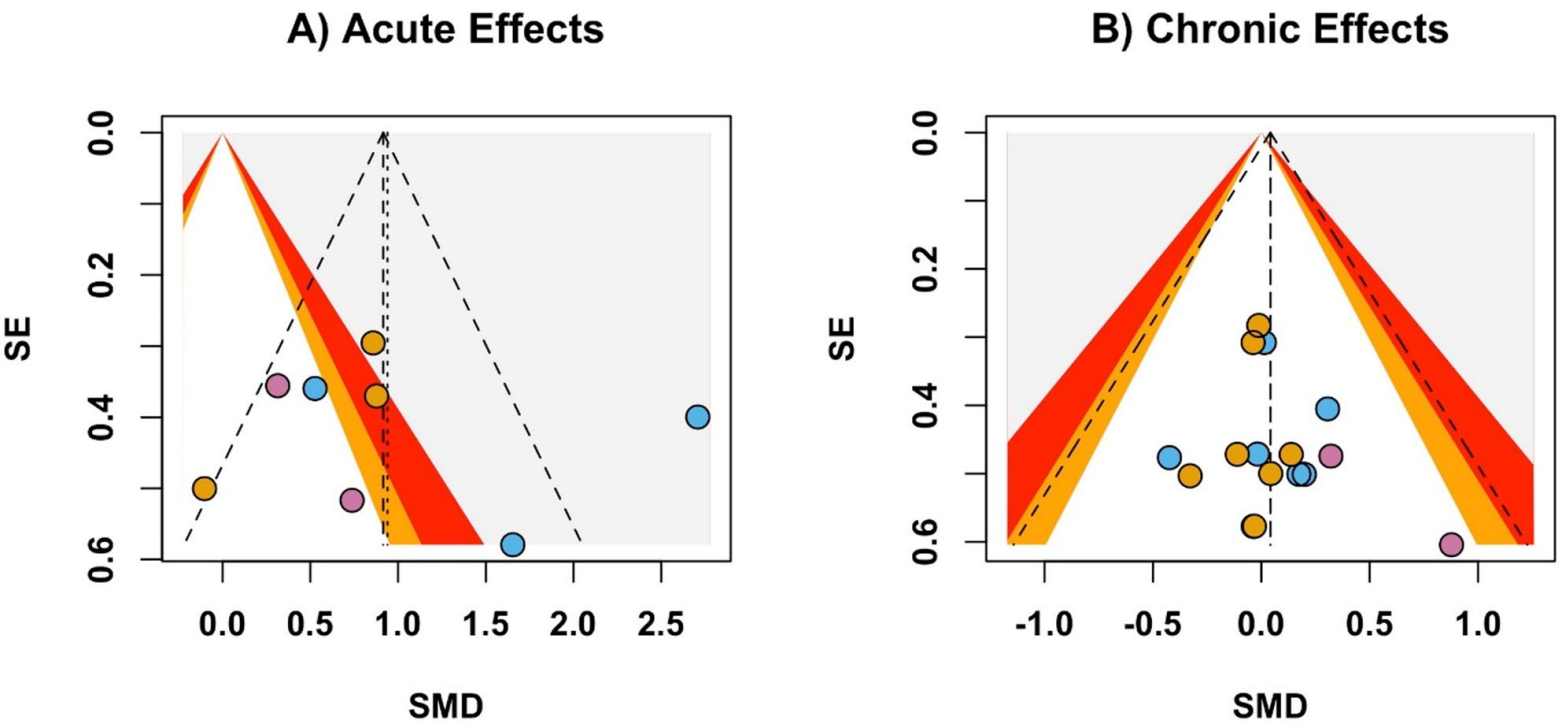
Funnel plots illustrating the relationship between effect size (standardized mean difference) and study precision (standard error) to assess potential publication bias for (**A**) acute responses and (**B**) chronic adaptations. In **A**, markers represent rating of perceived exertion (blue), blood lactate (orange), and heart rate (red). In **B**, markers represent muscle hypertrophy (blue), muscular strength (orange), and muscular endurance (red). The vertical dashed line represents the pooled effect size, while the triangular ‘funnel’ indicates the expected distribution of studies in the absence of publication bias. *SMD* standardized mean difference, *SE* standard error

For acute effects, 8 comparisons from 4 trials were included (Fig. 2). The meta-analysis revealed a large increase in RPE (fixed effect SMD 1.53, 95% CI 1.05 to 2.01, *p* < 0.001; random effects SMD 1.62, 95% CI 0.33 to 2.91, *p* = 0.014; Fig. 2A) and lactate (fixed effect SMD 0.70, 95% CI − 0.28 to 1.11, *p* < 0.001; random effects SMD 0.67, 95% CI 0.20 to 1.14, *p* = 0.006; Fig. 2B) for DROP compared with TRAD. In contrast, small and statistically non-significant effects were observed for heart rate (fixed and random effects SMD 0.45, 95% CI − 0.12 to 1.02, *p* = 0.124; Fig. 2C). The funnel plot for acute outcomes displayed a symmetric distribution (Fig. 3A), suggesting no relevant publication bias. This was supported by Egger’s regression test, which indicated no evidence of funnel plot asymmetry (*p* = 0.842). Thus, no trim-and-fill adjustments were required. Heterogeneity differed across outcomes, with considerable heterogeneity for RPE (I² = 87.9%), low heterogeneity for lactate (I² = 35.4%), and no heterogeneity for heart rate (I² = 0.0%).

### Meta-analytical Findings on Chronic Adaptations

**Fig. 4:**
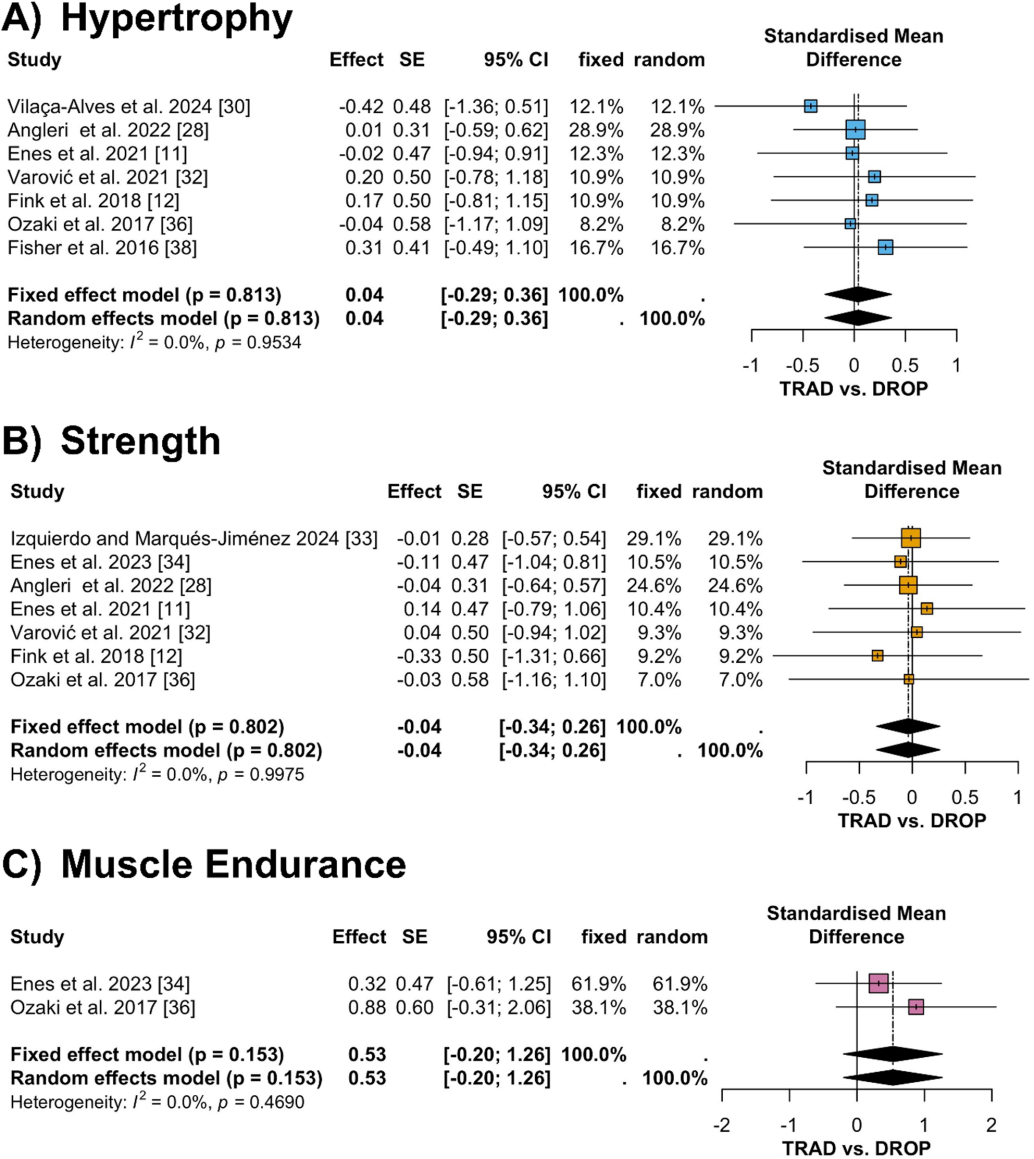
Forest plots illustrating the long-term adaptations in muscle hypertrophy, muscular strength, and muscular endurance, expressed as standardized mean differences and 95% confidence intervals, comparing drop-set and traditional resistance training. Panels represent (**A**) muscle hypertrophy, **B** muscular strength, and **C** muscular endurance. *CI* confidence interval, *DROP* drop-set training, *SMD* standardized mean difference, *TRAD* traditional resistance training

The meta-analysis for chronic strength, local muscle endurance and hypertrophy outcomes included 16 comparisons nested within 9 studies (Fig. 4). The pooled effects were trivial and not statistically significant across these outcomes, with hypertrophy showing a pooled SMD of 0.04 (95% CI − 0.29 to 0.36, *p* = 0.813, Fig. 4A), strength showing a pooled SMD of − 0.04 (95% CI − 0.34 to 0.26, *p* = 0.802, Fig. 4B), and local muscle endurance showing a pooled SMD of 0.53 (95% CI − 0.20 to 1.26, *p* = 0.153, Fig. 4C). Fixed effect and random effects models yielded identical pooled estimates for all three outcomes. Visual inspection of the funnel plot (Fig. 3B) for the chronic outcomes showed a symmetric distribution, indicating minimal publication bias. This was supported by Egger’s regression test, which indicated no evidence of funnel plot asymmetry (*p* = 0.428). Thus, no trim and fill adjustments were required. Heterogeneity was low, as indicated by low I² values. Sensitivity analyses confirmed the robustness of the results, with no significant changes observed when individual studies were excluded.

Subsequent subgroup analysis revealed that both untrained (SMD = − 0.05, 95%CI [− 0.37 to 0.28], *p* = 0.661, I^2^ = 0%) and trained (SMD = 0.01, 95%CI [− 0.73 to 0.75], *p* = 0.929, I^2^ = 0%) participants had only trivial chronic hypertrophy effects for the DROP vs. TRAD comparison. Similarly, both the volume-equivalent (SMD = − 0.05, 95%CI [− 0.42 to 0.32], *p* = 0.900, I^2^ = 0%) and non-volume-equivalent (SMD = − 0.02, 95%CI [− 0.51 to 0.48], *p* = 0.950, I^2^ = 0%) DROP vs. TRAD comparisons showed only trivial chronic hypertrophy effects. Regarding the different hypertrophy measurement approaches, muscle thickness (SMD = − 0.09, 95%CI [− 0.64 to 0.46], *p* = 0.655, I^2^ = 0%), muscle cross-sectional area (SMD = 0.04, 95%CI [− 0. 43 to 0.51], *p* = 0.953, I^2^ = 0%) and lean body mass (SMD = 0.31, 95%CI [− 0.49 to 1.10], *p* = 0.950, I^2^ = 0%) showed trivial to small chronic hypertrophy effects for DROP compared to TRAD. Similar to hypertrophy, both untrained (SMD = − 0.05, 95%CI [− 0.37 to 0.28], *p* = 0.974, I^2^ = 0%) and trained (SMD = 0.01, 95%CI [− 0.73 to 0.75], *p* = 0.922, I^2^ = 0%) participants showed only trivial chronic strength effects for DROP vs. TRAD. Similarly, both the volume-equivalent (SMD = − 0.05, 95%CI [− 0.42 to 0.32], *p* = 0.972, I^2^ = 0%) and non-volume-equivalent (SMD = − 0.02, 95%CI [− 0.51 to 0.48], *p* = 0.975, I^2^ = 0%) DROP versus TRAD comparisons showed only trivial chronic strength effects. Furthermore, both lower (SMD = − 0.01, 95%CI [− 0.33 to 0.32], *p* = 0.997, I^2^ = 0%) and upper body (SMD = − 0.20, 95%CI [− 0.94 to 0.54], *p* = 0.699, I^2^ = 0%) outcome exercises showed only trivial effects for the DROP versus TRAD comparisons.

### Systematic Review Approach

Beyond the meta-analytic approach, two studies investigated psychophysiological responses following DROP and TRAD [31, 35]. Enes et al. [31] examined the subjective feeling of pleasure/ displeasure during training and found that DROP resulted in significantly lower pleasure ratings (i.e., higher displeasure scores) for the back squat, 45° leg press, and seated knee extension compared to TRAD. Overall, DROP was perceived as significantly more unpleasant than TRAD (*p* < 0.001, d = 0.86). Similarly, Silva et al. [35] assessed pain perception using the Borg pain scale before, immediately after, and 24 h post-exercise. Their findings indicated that pain perception was significantly higher in DROP at the end of the session compared to TRAD, but this difference disappeared after 24 h.

Three studies analyzed energy demands and metabolic responses [29, 37, 40]. Goto et al. [40] investigated acute changes in lactate response and thigh girth (i.e., “muscle pump”) following two modified DROP protocols. The protocol consisted of five sets of TRAD (90% 1RM), followed by a 30-s rest period and a drop set using 50% or 70% 1RM. Both the 50% and 70% 1RM conditions led to significantly elevated lactate levels (50% 1RM: 4.3 ± 0.3 mmol/l; 70% 1RM: 3.8 ± 0.3 mmol/l [SEM]) and increased thigh girth (50% 1RM: 4.8 ± 0.4%; 70% 1RM: 3.1 ± 0.2% [SEM]) compared to baseline. Enes et al. [37] assessed aerobic and anaerobic energy expenditure during a bench press protocol. DROP elicited significantly greater aerobic energy expenditure than TRAD (*p* = 0.002; Hedges' g = 0.95), with trends toward higher absolute and relative aerobic expenditure and increased V·O2 values. However, no significant differences were observed in anaerobic energy expenditure. Furthermore, Angleri et al. [29] utilized near-infrared spectroscopy to analyze microvascular oxygenation of the vastus medialis. Their findings showed significantly lower levels of oxygenated hemoglobin and hemoglobin difference in DROP, accompanied by a significantly higher accumulation of deoxygenated hemoglobin.

Four studies evaluated the neuromuscular responses [12, 16, 29, 40]. Costa et al. [16] found a significant decline in countermovement jump performance from pre- to post-exercise only in the DROP condition (*p* < 0.001, 95% CI = 1.1 to 3.0, Δ% = − 6.7%, ES = 0.70), with post-intervention countermovement jump scores being significantly lower in DROP than in TRAD (*p* = 0.001, 95% CI = − 3.1 to − 0.7). Similarly, peak force significantly declined following DROP (*p* < 0.001, 95% CI = 9.3 to 25.2, Δ% = − 3.8%, ES = 0.27), with significantly lower post-intervention values in DROP compared to TRAD (*p* = 0.01, 95% CI = − 27.8 to − 3.1). Additionally, peak power significantly declined from pre- to post-training in DROP, whereas TRAD maintained peak power. Fink et al. [12] reported a significant reduction in maximal voluntary isometric contraction of the triceps brachii following DROP (*p* = 0.01, 95% CI = − 27.8 to − 3.1), which was not observed in TRAD. Goto et al. [40] corroborated these findings, reporting reductions in maximal voluntary isometric contraction ranging from 11.6% to 18.1% across various DROP protocols compared to only 6.3% in TRAD. In contrast, Angleri et al. [29] found no significant differences in muscle activation (EMG amplitude) of the vastus medialis between conditions.

Regarding long-term responses, Enes et al. [34] examined psychophysiological responses in resistance-trained men over eight weeks of DROP and TRAD. They assessed session RPE, training monotony and training strain (derived from session RPE) 15 min post-exercise after each training session. They found no significant differences between DROP and TRAD for any of the measures. Additionally, Goto et al. [39] compared a 4-week modified DROP protocol to TRAD following a 6-week lead-in period (see Table 1 for specifics). While no significant differences were observed for thigh cross-sectional area or MVIC, muscular endurance (total work volume in knee extensions) improved significantly more in the DROP group (+ 18.8 ± 9.4% [SEM] compared to TRAD (− 4.7 ± 5.8% [SEM]).

## Discussion

This study provides a comprehensive analysis of the acute and chronic effects of DROP compared to TRAD. By synthesizing findings from recent literature, we addressed existing gaps regarding acute metabolic and perceptual responses, alongside long-term adaptations. Our meta-analysis demonstrates that DROP elicits significantly higher acute ratings of perceived exertion and blood lactate concentrations than TRAD, whereas pooled differences in heart rate were not statistically significant.

### Acute Responses to Drop-Set Training

Our outcome-specific meta-analyses indicate that DROP is consistently associated with higher perceived exertion than TRAD, as well as a greater pooled effects for blood lactate concentration (Fig. 2B). In contrast, pooled differences in heart rate (Fig. 2C) did not reach statistical significance. Accordingly, large and statistically significant effects were observed for RPE and lactate, whereas confidence intervals for heart rate crossed the null, indicating no consistent differences between conditions based on the currently available evidence.

Although pooled analyses revealed significantly higher post-exercise lactate concentrations following DROP, individual study findings remain heterogeneous. Fink et al. [12] reported a non-significant difference in blood lactate kinetics; while TRAD peaked immediately post-exercise (409.9 ± 316.0% compared to baseline), DROP peaked at 2 min (313.2 ± 136.3%). Similarly, da Silva et al. [35] found no statistical differences in lactate immediately post-set, despite descriptively higher values in DROP (9.1 ± 1.9 mmol/l vs. 7.2 ± 2.4 mmol/l). Conversely, Enes et al. [37] and Goto et al. [40] reported significantly elevated lactate concentration post-exercise in DROP. Taken together, while pooled analyses indicate significantly higher blood lactate concentrations following DROP, variability between individual studies and differences in sampling time points warrant cautious interpretation of the magnitude of this effect.

Despite the lack of pooled significance, we observed a non-significant tendency toward higher heart rate in DROP (SMD 0.45, 95% CI − 0.12 to 1.02; Fig. 2C) [12, 35]), accompanied by lower oxygenated hemoglobin, higher deoxygenated hemoglobin [29], higher RPE [12, 31, 35], and increased pain perception [35]. The results indicate that the DROP structure imposes a more significant metabolic challenge, necessitating repetitions to failure with limited rest between consecutive load reductions [12, 29, 37]. In conjunction with the observed lactate responses, this metabolic stress likely reflects a greater reliance on anaerobic glycolysis, whereby elevated lactate concentrations [35, 37, 40] and associated hydrogen ion accumulation may exacerbate muscle fatigue and impair subsequent force production [42].

Notably, DROP experienced significantly higher RPE, indicating greater perceived exertion and discomfort during training [31, 35]. However, a longitudinal comparison over 8 weeks found that session RPE averaged across training sessions did not differ between groups [34]. This discrepancy may arise because session RPE assesses whole-session effort, whereas acute RPE captures the peak discomfort of individual sets [43, 44]. Furthermore, the absence of inter-set recovery in DROP likely exacerbates increased neuromuscular fatigue [11, 12, 16, 40]. Indeed, Costa et al. [16] and Fink et al. [12] reported significant reductions in neuromuscular performance measures such as countermovement jump, peak force, peak power, and maximal voluntary isometric contraction after DROP compared to TRAD, reinforcing the impact on neuromuscular fatigue.

Taken together, the acute evidence most consistently supports higher perceived exertion, greater neuromuscular fatigue, and elevated blood lactate concentrations following DROP, whereas cardiovascular responses, such as heart rate, appear less consistent at the pooled level. While this heightened exertional and metabolic demand may enhance training efficiency, it may also compromise training adherence, particularly among less experienced individuals or those engaging in high-frequency resistance training programs.

### Chronic Effects of Drop-Set Training

Our meta-analysis suggests that DROP and TRAD yield comparable hypertrophic, strength, and muscle endurance adaptations over time, despite their fundamental differences in execution. Although heterogeneity for the chronic outcomes was low in the present analyses, we retained a random effects framework a priori, and also computed fixed effect models. As expected when between study variance is negligible, both models yielded virtually identical pooled estimates. Both methods led to significant increases in muscle size, strength, and endurance, reinforcing the principle that sufficient intensity and volume are key drivers of neuromuscular adaptations [3, 45]. These findings are consistent with previous meta-analyses showing that mechanical tension and metabolic stress are the primary stimuli for muscle growth and are effectively induced by both training modalities [14, 15].

Although hypertrophic and strength outcomes between DROP and TRAD were statistically similar, DROP has consistently demonstrated superior time efficiency, often requiring half to one-third of the training duration of TRAD [14]. This advantage is particularly relevant for athletes and individuals with time constraints, potentially allowing for higher training frequency or additional exercises within a session. However, the greater neuromuscular fatigue associated with DROP must be considered, as it may necessitate longer recovery periods, potentially influencing training frequency and total workload [12].

Interestingly, while not statistically significant at the meta-analytical level, the SMD for muscular endurance favored DROP (0.53, 95% CI [-0.20; 1.26]). This is supported by Goto et al. [39], who reported a significant increase in muscular endurance after 4 weeks of DROP (+ 18.8 ± 9.4% [SEM]) compared to TRAD (− 4.7 ± 5.8% [SEM]). This suggests that DROP may enhance endurance by requiring the execution of submaximal repetitions in a state of high metabolic fatigue. However, the atypical nature of some protocols [39, 40], which included longer rest periods after multiple regular RT sets before a final drop set, limits broad conclusions.

Taken together, on a meta-analytic scale, both protocols led to muscle hypertrophy, strength, and muscle endurance improvements. Given that the average study duration in our analysis was 9.1 ± 2.2 weeks (range: 6–12 weeks), it remains uncertain whether the higher acute fatigue and perceived exertion from DROP could impair long-term hypertrophic, strength, or endurance adaptations over extended periods.

### Mechanisms of Muscular Adaptations in Drop-Set Training

The primary drivers of resistance training-induced hypertrophy include mechanical tension, metabolic stress, and muscle damage, although mechanical tension remains the most important factor [3, 5, 46]. While TRAD emphasizes mechanical tension by maintaining heavier loads across multiple sets, DROP provides a unique stimulus by combining sustained mechanical tension with high metabolic demand [12]. The potential mechanisms through which DROP may promote hypertrophy include increased time under tension, metabolic stress-induced signaling pathways, and enhanced motor unit recruitment. Time under tension plays a crucial role in muscular adaptation, as prolonged contractions lead to sustained activation of motor units and increased recruitment of muscle fibers [47]. By extending the duration of muscular contractions within a single set, DROP may enhance the activation of anabolic signaling pathways, particularly the mTORC1 pathway, which regulates muscle protein synthesis [48]. This extended activation period may be one of the key factors explaining why DROP can achieve similar hypertrophic outcomes despite the use of progressively lighter loads. In addition to time under tension, metabolic stress has been proposed as a contributor to hypertrophy [3, 5]. The accumulation of metabolites such as lactate, inorganic phosphate, and hydrogen ions during DROP may stimulate muscle growth through mechanisms such as cellular swelling, hypoxia-induced adaptations, and hormonal responses [40, 49]. While acute hormonal spikes (e.g., growth hormone) were traditionally linked to these metabolites [6, 40], their actual influence on long-term hypertrophy is now questioned [50, 51]. Importantly, these mechanistic pathways should be interpreted as plausible but not definitively confirmed in this context. Another factor contributing to hypertrophy in DROP is the recruitment of muscle fibers. Performing repetitions to failure ensures the recruitment of high-threshold motor units according to Henneman’s size principle [52, 53], ensuring both type I and type II fiber activation despite the use of progressively lighter loads. The high degree of fiber recruitment observed in DROP may partially explain why hypertrophy outcomes are similar to those of TRAD, despite differences in loading strategies.

Importantly, while the greater acute metabolic stress and fiber recruitment associated with DROP may provide an effective hypertrophic stimulus, they also result in increased neuromuscular fatigue. This fatigue could impact training frequency and overall recovery capacity [12]. This observations aligns with previous research suggesting that frequent training to failure increases recovery demands, potentially leading to diminished performance in subsequent sessions [54]. Consequently, while DROP is a time-efficient strategy for stimulating muscle growth, strength, and muscle endurance, its integration into a long-term program requires careful implementation.

### Limitations and Future Research Perspectives

While this meta-analysis provides valuable insights into the acute and chronic effects of DROP, several limitations must be acknowledged. One of the primary constraints is the heterogeneity of the included studies, particularly in terms of participant characteristics, training experience, and protocol design. Differences in training frequency, exercise selection, and load reduction strategies could introduce variability in the observed results, limiting the generalizability of our findings. For instance, some studies implemented a single drop set, whereas others employed multiple drop sets or modified versions DROP [39, 40], which may impact hypertrophic and strength adaptations differently. Standardizing drop-set methodologies in future research would make results more comparable and allow more precise conclusions to be drawn about their effectiveness.

Beyond protocol heterogeneity, the inherent methodological limitations of resistance training research must be considered. While the PEDro scale is a recognized and valid instrument for assessing the methodological quality of clinical trials [19, 55], it is essential to recognize its inherent boundaries within the field of exercise science. A primary constraint is that double-blinding (PEDro items 5–7) is practically impossible to implement in resistance training interventions. This structural limitation often caps the maximum achievable scores for even the most rigorous trials, which should not be misinterpreted as a lack of scientific quality. Furthermore, the scale does not account for the impact of the small sample sizes observed in this review, with an average of 21 ± 8 participants. These small sample sizes can significantly reduce statistical power and increase the risk of type II errors when comparing DROP and TRAD.

Another limitation is the lack of long-term investigations on the sustainability of DROP adaptations, particularly in trained individuals and athletic populations. Most studies included in our analysis examined hypertrophic and strength outcomes over relatively short durations, typically between 6 and 12 weeks. Given that resistance training adaptations are cumulative and periodization strategies vary across athletic and clinical contexts, further research is needed to determine whether drop-set training remains an effective stimulus for muscle growth and strength development over extended training periods. Additionally, the long-term impact of increased neuromuscular fatigue associated with drop sets on recovery dynamics, injury risk, and overall training progression remains largely unexplored. Future studies should employ longitudinal designs with follow-up assessments to assess whether drop sets are sustainable within structured training cycles.

## Practical Applications

The findings of this meta-analysis highlight several practical implications for athletes, coaches, and recreational trainees seeking to optimize hypertrophic and strength adaptations through time-efficient training strategies. One of the most notable advantages of drop-set training is its ability to achieve comparable muscle hypertrophy and strength gains to traditional resistance training while requiring significantly less training time. This efficiency makes drop sets particularly attractive for individuals with time constraints, such as competitive athletes with high overall training loads, individuals balancing fitness with professional obligations, or clinical populations undergoing rehabilitation programs where session duration is a limiting factor. Despite its benefits, drop-set training should be implemented strategically rather than as a wholesale replacement for traditional resistance training. Given that DROP elicits greater rating of perceived exertion and neuromuscular fatigue, it may be best suited for specific training phases or muscle groups rather than for full-body training across all sessions. Additionally, given the heightened perceived exertion associated with drop sets, their use in higher-frequency training programs should be carefully managed to avoid excessive fatigue accumulation that may impair recovery and subsequent performance. Our key results are summarized in Table 2.

**Table 2:** Summary of results

Variable	DROP vs. TRAD
Acute effects	
Rating of perceived exertion	△ Higher in DROP
Lactate	△ Higher in DROP
Heart rate	No difference
Neuromuscular fatigue	△ Greater in DROP
Aerobic energy expenditure	△ Greater in DROP
Feeling of displeasure	△ Greater in DROP
Pain scale	△ Greater in DROP
Chronic effects	
Hypertrophy	No difference
Strength	No difference
Muscle endurance	No difference

*DROP* drop-set training, *TRAD* traditional resistance training

## Conclusion

This meta-analysis provides a comprehensive evaluation of the acute and chronic effects of DROP compared to TRAD. The findings demonstrate that DROP is an effective and time-efficient training method, capable of eliciting similar hypertrophic and strength adaptations as TRAD while requiring significantly less training time. The key distinguishing factor between the two methods lies in the greater acute metabolic stress and neuromuscular fatigue induced by DROP, which, while beneficial for maximizing training efficiency, also necessitates careful integration into structured resistance training programs. However, the acute evidence is outcome specific, with the most robust pooled difference observed for perceived exertion, while lactate and heart rate did not show statistically significant pooled differences. Therefore, conclusions about heightened acute physiological stress should be framed cautiously and should not rely on a single subjective marker alone. While drop-set training offers clear advantages in terms of efficiency, several research gaps remain, particularly regarding its long-term sustainability, effects on different muscle fiber types, and interactions with recovery strategies. Given its potential as a time-efficient resistance training strategy, further refinement of programming recommendations will allow athletes, coaches, and practitioners to better harness the benefits of drop-set training while minimizing its potential drawbacks.

## Data Availability

Not applicable.
